# GDNF-mediated rescue of the nigrostriatal system depends on the degree of degeneration

**DOI:** 10.1038/s41434-018-0049-0

**Published:** 2018-12-07

**Authors:** Luis Quintino, Martino Avallone, Emil Brännstrom, Patrick Kavanagh, Marcus Lockowandt, Patricia Garcia Jareño, Ludivine S Breger, Cecilia Lundberg

**Affiliations:** CNS Gene Therapy, Department of Experimental Medical Science, Lund University, Lund, Sweden

**Keywords:** Neurotrophic factors, Neurological disorders

## Abstract

Glial cell-line derived neurotrophic factor (GDNF) is a promising therapeutic molecule to treat Parkinson’s disease. Despite an excellent profile in experimental settings, clinical trials testing GDNF have failed. One of the theories to explain these negative outcomes is that the clinical trials were done in late-stage patients that have advanced nigrostriatal degeneration and may therefore not respond to a neurotrophic factor therapy. Based on this idea, we tested if the stage of nigrostriatal degeneration is important for GDNF-based therapies. Lentiviral vectors expressing regulated GDNF were delivered to the striatum of rats to allow GDNF expression to be turned on either while the nigrostriatal system was degenerating or after the nigrostriatal system had been fully lesioned by 6-OHDA. In the group of animals where GDNF expression was on during degeneration, neurons were rescued and there was a reversal of motor deficits. Turning GDNF expression on after the nigrostriatal system was lesioned did not rescue neurons or reverse motor deficits. In fact, these animals were indistinguishable from the control groups. Our results suggest that GDNF can reverse motor deficits and nigrostriatal pathology despite an ongoing nigrostriatal degeneration, if there is still a sufficient number of remaining neurons to respond to therapy.

## Introduction

Glial cell-line derived neurotrophic factor (GDNF) is a promising therapeutic agent to treat Parkinson’s disease (PD). Delivery of GDNF or GDNF family ligands (GFLs) to the striatum protects, regenerates, and improves the metabolism of substantia nigra pars compacta neurons (SNpc), a key neuronal population degenerating during PD pathogenesis [1–6], resulting in amelioration of motor deficits in PD models. The potent therapeutic effects of GFL resulted in the development of clinical trials to test if GDNF or GFL such as Neurturin can modify PD progression. Despite initial promise [7–10], large-scale clinical trials of GDNF and Neurturin have not been successful [5, 6, 11, 12] in rescuing the nigrostriatal system and improving motor deficits. Why did these clinical trials fail?

One theory postulates that the GDNF pathway is compromised in SNpc neurons of PD patients due to alpha-synuclein pathology [13] and for that reason, PD patients do not respond to GDNF treatment. Although this theory is supported by experimental data, a recent systematic study analyzing brains from PD patients, together with several animal models of PD, did not find any impairments in the GDNF pathway [14], suggesting that the deficits seen previously may be due to the specific experimental model used [13, 15]. Another theory proposes that drug delivery protocols were suboptimal, resulting in limited distribution of neurotrophic factors in the brain parenchyma that prevented the possibility of observing a therapeutic benefit [16]. Analysis of catheters and infusion protocols used in the GDNF trials, experimental studies testing GDNF delivery protocols in primates [17–19] as well as brains from patients that participated in the Neurturin clinical trial [16] seem to support this theory. The last theory proposes that the nigrostriatal degeneration in patients selected for clinical trials may have been too advanced for the patients to respond to GFL therapy [16]. This theory is supported by two sets of data. The first comes from the Neurturin trial, where stronger motor recovery was seen in patients with <5 years of disease [20]. The second comes from a study from Kordower et al. [21], analysis of brains from PD patients indicated that SNpc cells and dopaminergic terminals in the putamen seem to be lost within the first 4–7 years of diagnosis.

We set out to test if the therapeutic benefits of GDNF on SNpc neurons and motor behavior are dependent on the degree of nigrostriatal degeneration. We observed that if GDNF was given when the nigrostriatal system was fully degenerated there was no recovery. To rescue the nigrostriatal system and motor deficits, GDNF needed to be present in the striatum when there were still a significant number of impaired SNpc neurons to respond to therapy.

## Results and discussion

### Experimental design

**Fig. 1:**
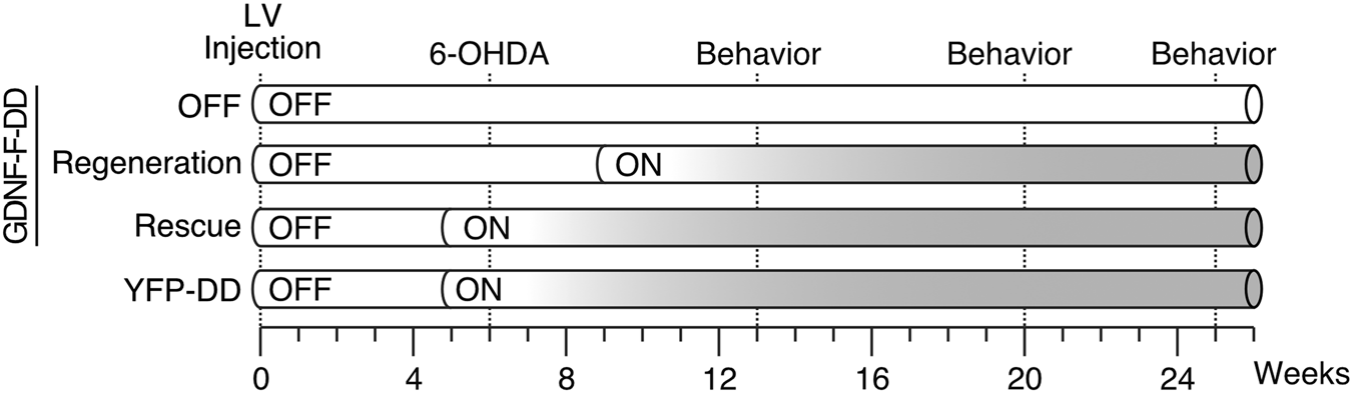
Experimental design. Six weeks after lentiviral vector delivery, the animals were lesioned with an intrastriatal 6-OHDA lesion protocol. Behavior assessment was performed 7, 14, and 19 weeks after lesion. The animals were euthanized 19 weeks after lesion. One subgroup of LV-GDNF-F-DD animals was used to test if GDNF could rescue SNpc cells and motor behavior from a degenerating dopaminergic system (Rescue). Another subgroup of LV-GDNF-F-DD animals was used to test if GDNF could regenerate SNpc cells and motor behavior after the nigrostriatal system has degenerated (Regeneration). One subgroup of LV-GDNF-F-DD animals was not given any TMP (OFF) and used as control for DD leakiness. The LV-YFP-DD animals, and Rescue and Regeneration animals were given TMP continuously 1 week before 6-OHDA lesion and used as lesion control

To determine if the therapeutic effects of GDNF are dependent on the degree of nigrostriatal degeneration, lentiviral vectors expressing destabilizing domain (DD)-regulated yellow fluorescence protein (LV-YFP-DD) or DD-regulated GDNF (LV-GDNF-F-DD) [22–24] were delivered to the striatum of rats and the experiment was performed as shown in Fig. 1. DD regulation was achieved by making a fusion protein of a protein of interest with a DD, a mutated degradation-prone protein or peptide. Due to the presence of the DD, the full fusion protein is targeted for proteasomal dagradation. The DD is designed so that proteasomal recognition could be blocked by a drug. The DD used in this study requires trimethoprim (TMP) to block proteasomal recognition [22–24].

The animals were lesioned with an intrastriatal 6-OHDA lesion protocol so that the vast majority of susceptible SNpc cells would die progressively within the first 4–5 weeks of lesion [25–27], providing a window of progressive degeneration to test the hypothesis that the benefits of GDNF on SNpc are dependent on the degree of nigrostriatal degeneration.

One subgroup of LV-GDNF-F-DD animals was used to test if GDNF could rescue the dopaminergic system from an ongoing degeneration (Rescue), modeling nigrostriatal pathology at time of PD diagnosis. As SNpc neurons need 2–3 weeks of continuous TMP treatment to respond to GDNF-F-DD [23] and the 6-OHDA degeneration occurs within 4–5 weeks, TMP treatment was started 1 week before 6-OHDA to allow SNpc neurons to respond to GDNF-F-DD induction when half of the SNpc neurons have died due to 6-OHDA but a significant number of impaired SNpc neurons remained to regenerate the nigrostriatal system.

Another subgroup of LV-GDNF-F-DD was used to test if GDNF could regenerate the nigrostriatal system after most of the SNpc neurons have died (Regeneration), modeling end-stage nigrostriatal pathology seen in advanced PD. TMP treatment was started 3 weeks after 6-OHDA lesion, therefore GDNF-F-DD activation of SNpc neurons reached maximum levels 5–6 weeks after lesion, when the vast majority of SNpc neurons have already died from 6-OHDA.

### GDNF can rescue SNpc neurons and motor function

**Fig. 2:**
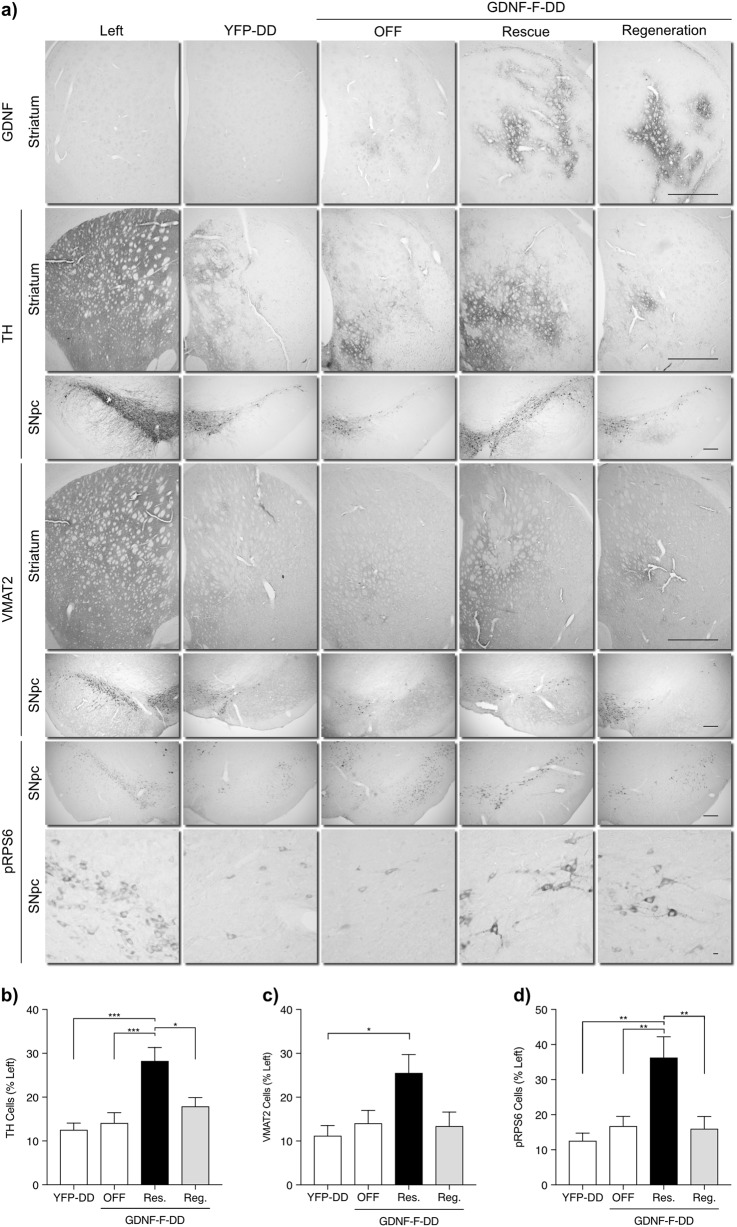
GDNF-F-DD is able to rescue the nigrostriatal system. **a** Immunohistochemical analysis for GDNF, TH, VMAT2, and pRPS6 was performed on brain from animals injected with LV-GDNF-F-DD that were treated with TMP during (Rescue) or after nigrostriatal degeneration (Regeneration). Control animals were injected with LV-GDNF-F-DD and given normal drinking water (OFF). Animals injected with LV-YFP-DD were also treated with TMP during nigrostriatal degeneration (YFP-DD). **b** Quantification of TH-positive cells in SNpc (*n* = 7–10 per group). **c** Quantification of VMAT2-positive cells in SNpc (*n* = 8–10 per group). **d** Quantification of pRPS6-positive cells in SNpc (*n* = 6–9 per group). One-way ANOVA with Tukey multiple comparison tests performed. *p ≤ 0.05, **p ≤ 0.01, ***p ≤ 0.0001. Large scale bar—1200 µm. Medium scale bar—200 µm. Small scale bar—10 µm

Immunohistochemistry for GDNF (Fig. 2a) showed expression in the Rescue and Regeneration groups and minimal staining for the OFF group. This is in line with previous observations and quantifications of GDNF-F-DD expression in vivo [22, 23].

Immunohistochemistry for tyrosine hydroxylase (TH) (Fig. 2a) indicated a severe striatal denervation in the YFP-DD, Regeneration, and OFF groups, which was ameliorated in the Rescue group. There was also a severe reduction of SNpc TH-positive neurons in all groups. Similarly, quantification of TH neurons in SNpc (Fig. 2b) confirmed a higher number surviving TH neurons in the Rescue group when compared to the remaining groups. One-way analysis of variance (ANOVA; *F* = 8.7, *p* = 0.0003) and subsequent post hoc test showed a statistically significant difference in TH-positive neurons between Rescue and all other groups.

Immunohistochemistry for vesicular monoamino transporter 2 (VMAT2) (Fig. 2a, c) indicated striatal denervation and SNpc degeneration levels similar to TH. The Rescue group seemed to have the strongest striatal VMAT2 staining and the highest number of SNpc VMAT2 neurons. One-way ANOVA analysis of VMAT2 neurons indicated differences between groups (*F* = 3.5, *p* = 0.02) and post hoc analysis confirmed a statistically significant difference in VMAT2-positive neurons between Rescue and YFP-DD groups. There was a higher variability in the VMAT2 neuron numbers when compared with TH neuron numbers that may explain why we could only detect a difference between the Rescue and YFP-DD groups.

SNpc sections were also stained for phosphorylated ribosomal protein S6 (pRPS6). This protein is downstream of Akt and Erk signaling pathways. RPS6 is phosphorylated in SNpc neurons due to GDNF-mediated activation of signaling pathways and for that reason has been used as a marker for GDNF activity in SNpc cells [13, 22, 23]. Similarly, to TH and VMAT2, there were increased numbers of pRPS6 neurons in the Rescue group when compared with the remaining groups (Fig. 2a, d). One-way ANOVA revealed statistically significant differences between groups (*F* = 8.2, *p* = 0.0006) and post hoc analysis indicated a difference in pRPS6 neuron numbers between the Rescue and all remaining groups.

Interestingly, pRPS6 immunohistochemistry also showed surviving SNpc neurons with more intense pRPS6 staining and increased perikarya size in the injected hemispheres of Rescue and Regeneration groups. According to the literature, increased pRPS6 cell numbers, stronger staining [13, 22], and increased SNpc neuron perikarya size [28, 29] suggest that these neurons were responding to GDNF. TH, VMAT2, and pRPS6 showed a very similar picture: that there was a therapeutic response of GDNF-F-DD in the Rescue group in contrast with the remaining groups.

**Fig. 3:**
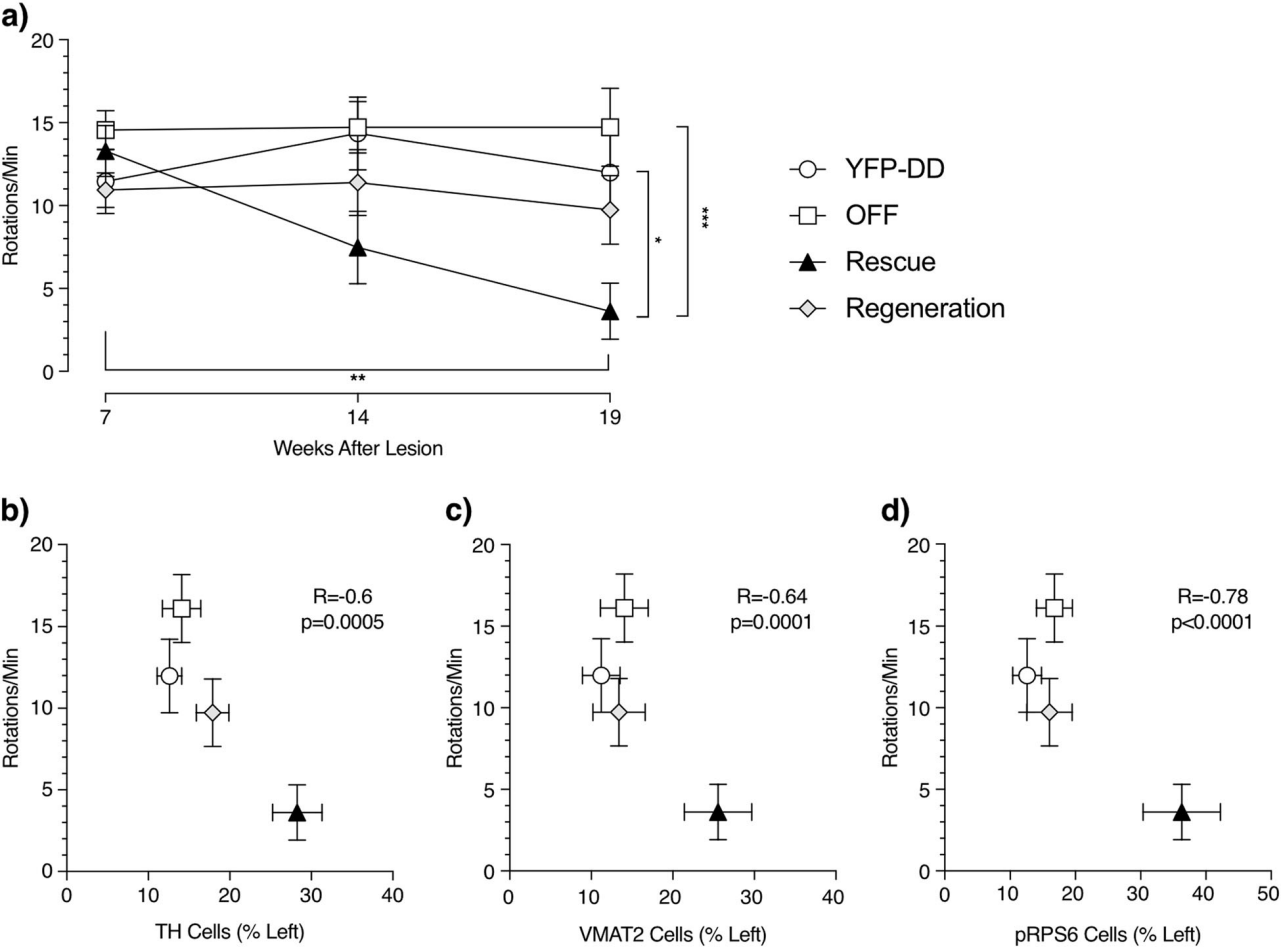
GDNF-F-DD leads to partial recovery of motor impairment when there is an ongoing nigrostriatal degeneration. **a** Amphetamine-induced rotations measured 7, 14, and 19 weeks after 6-OHDA lesion (*n* = 8–10 per group). One-way ANOVA with Tukey multiple comparison tests performed on week 19. Repeated-measures ANOVA with Tukey multiple comparison tests performed on weeks 7, 14, and 19 was statistically significant in the Rescue group. *p ≤ 0.05, **p ≤ 0.01, ***p ≤ 0.0001. **b** Correlation between numbers of TH neurons and amphetamine-induced rotations on week 19. **c** Correlation between numbers of VMAT2 neurons and amphetamine-induced rotations on week 19. **d** Correlation between numbers of pRPS6 neurons and amphetamine-induced rotations on week 19

Assessment of motor deficits was done by amphetamine-induced rotations (Fig. 3a). At 7 weeks after lesion, all groups exhibited a strong rotational bias, indicative of severe nigrostriatal degeneration. Interestingly, 14 and 19 weeks after lesion, the Rescue group showed a progressive decrease in the number of ipsilateral rotations, suggesting that a rescue of nigrostriatal degeneration was taking place. In contrast, the rotational bias levels were constantly high at all time points for Regeneration, YFP-DD, and OFF groups. Repeated-measures ANOVA showed that only the Rescue group showed a statistically significant reduction of amphetamine-induced rotational behavior (*F* = 14.3, *p* = 0.001) between weeks 7 and 19 after lesion. In addition, one-way ANOVA comparing the rotational bias of the different groups on week 19 indicated differences between groups (*F* = 6.6, *p* = 0.002). Subsequent post hoc analysis showed differences between the Rescue, YFP-DD, and OFF groups. The motor assessment suggested that there was therapeutic response of GDNF-F-DD in the Rescue group in contrast with the remaining groups.

Correlation analysis comparing the cell counts and rotational bias at week 19 after lesion showed a significant reverse correlation (Fig. 3b–d). When the individual values were sorted by experimental group, it became clear that the animals from the Rescue group clustered apart from the remaining groups, with negligible differences between Regeneration, OFF, and YFP-DD groups.

The behavior and histological data indicate that our results are not due to neuroprotection from GDNF-F-DD. We observed no protection of motor impairments at 7 weeks after lesion, nor did we observe a high number of surviving SNpc cells. These are two key aspects observed when viral vectors expressing GDNF are given in a neuroprotective paradigm (i.e. SNpc neurons respond to GDNF before the lesion): there is a higher number of surviving SNpc neurons and a significant protection of motor impairment already 7 weeks post lesion [30–36]. When we previously tested GDNF-F-DD for neuroprotection, and in contrast with the current study, we observed a significantly higher number of surviving SNpc neurons and a significant protection from motor impairments already 6 weeks after lesion.

The data suggest instead that the GDNF-F-DD therapeutic response was due to a rescue of impaired SNpc cells. Our study is in accordance with literature showing that GDNF can rescue an impaired nigrostriatal system. In studies where GDNF protein [29, 37–40] or viral vectors expressing GDNF [41–45] were given 4 weeks after 6-OHDA lesion, there was a milder lesion that resulted in 20–40% living SNpc neurons in control groups. We observed 10% surviving neurons in the YFP, Regeneration, and OFF groups. Thus, the GDNF groups in these previous studies are comparable to the Rescue group where there was still a critical number of surviving SNpc neurons that enabled rescue of the nigrostriatal system. Moreover, when GDNF was given 2 weeks after 6-OHDA, mimicking the situation in the Rescue group, the level of motor recovery and nigrostriatal pathology was similar [46] to what we have observed.

Our study is the first directly comparing the response of the nigrostriatal system to GDNF in two important conditions: during ongoing degeneration and/or in a fully degenerated nigrostriatal system. The results indicate that for GDNF to be effective, it needs to be given when sufficient SNpc neurons are still present. Below a certain threshold, the surviving neurons will respond to GDNF but their numbers will not allow a rescue of nigrostriatal pathology.

The regulated GDNF-F-DD we have developed makes it possible to design studies to fine-tune GFL therapies. For example, it will allow us to pinpoint where the threshold for GDNF-mediated recovery is, as well as allow us to determine for how long the GDNF-mediated recovery of the nigrostriatal system can be maintained.

Importantly, the current study supports the idea that the GDNF/GLF clinical trials need to target a cohort of PD patients that is as close as possible to the time of diagnosis in order for the GDNF/GFL therapy to be successful. In addition to selecting less advanced patients, the poor distribution of Neurturin in the putament of patients in the CERE-120 trial also highlited the need for better delivery protocols and vector technologies [16]. If predictive biomarkers for PD can be developed, it will be possible in the future do provide GFL therapies to patients where the nigrostriatal pathology is still at subclinical levels, thereby maximizing the recovery of the nigrostriatal system.

## Materials and methods

### Viral vector production

Plasmids, cell culture conditions, and viral vector production details have been described in detail elsewhere [22, 23]. The LV had the functional titers of 1 × 10^9^ transducing units/ml.

### Animal experiments

All animals were housed and handled according to European and Swedish laws. All procedures have been approved and performed according to the guidelines established by the Ethical Committee for Use of Laboratory Animals at Lund University under the permit M366-12. A total of 40 Female Sprague Dawley rats, 10 per experimental group (Charles River, Sulzfeld, Germany), weighing 225–250 g were used for the experiments. 6-OHDA surgeries have been described in detail elsewhere [22]. Briefly, the 6-OHDA dosage was 2 × 10 µg of free-base 6-OHDA diluted in 3.5 µg/µl, delivered to the following coordinates with the tooth bar set at 0: (1) anteroposterior (AP) +0.5 mm, mediolateral (ML) −2.5 mm, dorsoventral (DV) −5 mm. (2) AP −0.5 mm, ML −4.2 mm, DV −5 mm. Viral vector surgeries have been described in detail elsewhere [22, 23]. Viral suspensions were delivered to three sets of coordinates: (1) AP +1.4 mm, ML −2.6 mm, DV −5/−4 mm. (2) AP +0.4 mm, ML −3.8 mm, DV −5/−4 mm. (3) AP −0.8 mm, ML −4.4 mm, DV −5/−4 mm. Tooth bar was set at 0 and a total of 6 µl of lentiviral vector suspension (1 µl/DV coordinate) was delivered at a rate of 0.4 µl/min. Perfusions were performed as described previously [22].

### TMP treatments

TMP (TMP oral suspension 10 mg/ml; Meda AB, Solna, Sweden) was diluted in water to 0.2 mg/ml and given to the animals in their drinking water continuously throughout the experiment.

### Behavioral assessment

Drug-induced rotations were performed using 2.5 mg/kg *d*-amphetamine sulfate over a period of 90 min as described previously [22].

### Histological analysis

Standard immunohistochemistry procedures have been described in detail elsewhere [22, 23]. The brains were cut into six series containing 35 µm-thick sections. For each staining, one series was used per animal. The samples were rinsed three times in 0.1 M potassium phosphate buffer saline (KPBS) and incubated for 15 min in quenching solution (KBPS, 10% methanol and 3% H_2_O_2_). The samples were then rinsed three times in KPBS and incubated for 1 h in 5% serum solution (KPBS + 5% horse or goat serum + 0.25% Triton X). Samples were then incubated overnight with primary antibody diluted in 5% serum solution. On the next day, samples were washed two times in KPBS, incubated for 15 min in 5% serum solution, and incubated for 1 h with secondary antibodies diluted in 5% serum solution. The samples were subsequently washed for three times with KPBS, incubated 1 h with KPBS containing ABC complex and washed further three times with KPBS. The samples were incubated with KPBS containing 0.5 mg/ml 3,3-diaminobenzidine for 2 min. The reaction was then visualized by incubating the samples with 10 µl H_2_O_2_ solution (KPBS + 0.9% H_2_O_2_) for 2–4 min. The samples were washed three times in KPBS, mounted, and coverslipped using DPX mounting medium. For the VMAT2 and pRPS6 staining, samples were incubated in Tris-EDTA Buffer (10 mM Tris base, 1 mM EDTA, and 0.05% Tween 20, pH 9) for 10 min at 80 °C before quenching. The following antibodies were used: rabbit anti pRPS6 (#2211, 1:2000, Cell Signaling Technology); goat anti-human GDNF (AF-212-NA, 1:1000, R&D Systems Europe); rabbit anti-TH (AB152, 1:1000, Merck); rabbit anti-VMAT2 (20042, 1:8000, Immunostar); biotinylated horse anti-mouse (BA2001, 1:200, Vector Labs, Peterborough, UK); biotinylated horse anti-goat (BA9500, 1:200, Vector Labs); and biotinylated goat anti-rabbit-biotin (BA1000, 1:200, Vector Labs).

### Quantification of cell numbers

Quantification of SNpc cell numbers has been described in detail elsewhere [22, 23]. Briefly, three coronal sections were used to quantify TH, VMAT2, pRPS6 positive SNpc neurons: the coronal section containing medial lemniscus separating ventral tegmental area from SNpc (approximately −5 mm relative to bregma), adjacent cranial section, and adjacent caudal section. Data were presented as a percentage of neurons relative to the left intact side.

### Statistics

Statistical analysis was performed using Graphpad Prism 7 (Graphed Software, La Jolla, CA). Figures show mean ± standard error of mean. When one-way ANOVA was performed, post hoc analysis was done using Tukey multiple comparison tests. When repeated-measures ANOVA was performed, post hoc analysis was done using Tukey multiple comparison tests.
